# The State Lunatic Asylum and Its Management

**Published:** 1887-08

**Authors:** 


F. E. DANIEL, M. D., EDITOR AND PUBLISHER.

COLLABORATORS:

*E. J. Doering, M. D., Chicago.*

*Geo. Cupples, M. D., San Antonio.*

*Odo Betz, M. D., Germany.*

*E. J. Beall, M. D., Fort Worth, Texas.*

*T. C. Osborn, M. D., Cleburne, Texas.*

*Wm. Penny, M. D., New York.*

*H. O. Marcy, M. D., Boston.*

*C. K. Gregg, M. D., Mexico.*

*R. M. Swearingen. M. D., Austin.*

*C. H. Wilkinson, M. D., Galveston.*

*J. W. McLaughlin, M. D., Austin.*

*R. H. L. Bibb, M. D., Mexico.*

### THE STATE LUNATIC ASYLUM, AND ITS MANAGEMENT.

Texas has reason to be proud of her splendid eleemosynary institutions. The magnificent structures and grounds of the several asylums, are pointed to with pride, and evidence a broad and comprehensive philanthropy—in keeping with the advanced civilization of the age. Strangers visiting the Capital are shown these institutions, as amongst the greatest of its attractions, and are impressed not only with the greatness of Texas, but with the goodness and humanity of her rulers. The princely appropriation too, each year made for their conduct is a matter of boast, and is highly creditable to the State. It *should be* equally a matter of pride that they are conducted with the highest degree of executive and administrative ability and professional skill, and with an eye single to the welfare of the unfortunate inmates, and the glory of the State, their generous patron.

That such has been the case with the School for the Blind, and the Asylum for the Deaf Mutes we have not heard questioned : but for some reason, the Lunatic Asylum has been a perpetual sore. Ever since we can remember, there has been some quarrel, or controversy, or complaint about the management of the Lunatic Asylum.

Now, this is a subject in which every citizen of Texas, and certainly, every physician, has an interest, and we propose to discuss it in a dispassionate way, and with prejudice towards none.

What is the reason for this state of affairs ? Is the management of this Institution a matter so difficult, that no one within the broad limits of Texas can be found competent to it? Does it require such a rare combination of qualities on the part of the Superintendent that few are equal to the task? By no means: The answer is quite plain. * * * *

Whatever else may be necessary on the part of a Superintendent, to a proper discharge of the duties—*self-control*, it seems to us, is an indispensable element. On general principles one would say that a man who cannot control his temper, and who will undertake, —no matter what may be the provocation,—to administer corporeal punishment with his own hands, to one of the employees, is *certainly* deficient in *one* of the requisites,—and is unfit for the discipline of the demented, the insane or the idiotic, as well as for the management of the business of the Institution. Leaving out of consideration the question of dignity, it is fair to assume that a superintendent who will allow himself to become involved in a personal wrangle with an employee, and so lose *his* head as to throw a spittoon at the other man's head—has gotten somehow in the wrong pew. This act, is, in itself, sufficiently disgraceful; but when the world reads—as they must have read in the telegrams sent over the wires all over the United States last week, that the Superintendent of the Texas State Lunatic Asylum had been *fined* in the Justice's Court, *for throwing a spittoon at the head of a nurse!* it is time for the eagle-bird that perches on the banner of the Lone Star State, to hang his head, and swap places with the blue jay.

But, as serious a matter as this is, the Honorable Board of Managers—all sensible and honorable men, have given it an indescribably *ludicrous* turn, by a well meant, (no doubt) but mistaken attempt to bolster up the Superintendent. Read the following.

SUPPORTING DR. DORSETT.

*To the Editor of the Statesman*:

At the regular monthly meeting of the board of managers, held yesterday at the State Lunatic Asylum, the following resolution was unanimously adopted:

"Resolved, That after a careful investigation of the case lately tried in Justice Tegener's court against Dr. Dorsett, the superintendent of this institution, for throwing a spittoon at the employe, George Ellis, the board is of the opinion that Dr. Dorsett was justifiable, and he is hereby exonerated from all blame for his action in the premises." OSCEOLA ARCHER,

President of the Board of Managers of State Lunatic Asylum.

Are there no "rules and regulations" for the government of employees? or do these rules and regulations comprehend "corporeal punishment", and direct that the superintendent shall administer it? Do they *prescribe the mode and manner* of its administration to be, and authorize, *the promiscous hurling of household furniture at offending heads?* We can not *conceive* of a *situation* where a dignified medical officer, on a large salary, in charge of a great charity, of a great State, founded for the *protection* and care of those entrusted to his keeping, could be "*justified*" in throwing a spittoon, or anything else, at one of the inmates. Mr. Archer, and his board, might have had their sympathies for Dr. D. so aroused by his account of the fracas—as to be lead to excuse him for a sudden burst of passion, in which he did a weak and foolish act— out of charity for all human weakness; but to "*justify*" him in hurling a spittoon at the head of a hireling, is the heighth of absurdity; besides, it is a dangerous precedent, and should be rebuked. What was the offence, that would "justify" such an act? No doubt, a very grave one; but it could not have been so grave as to be beyond remedy. The superintendent is almost an autocrat; he could have *called a guard*, and had the offender confined; he could have easily procured his discharge, or, if necessary, had him properly punished for his insubordination—if insubordination were the offence. We say dangerous, because, an offence a *little more aggravating* might "justify" the hurling of a looking glass— or a bed pan, or a bedstead, or a cooking stove, or any other article of furniture, at the offender;– or, one of the inmates might be seized by the leg by the irate superintendent, and "everlastingly worn out" over the back of the unruly "sub"; and, again,—if his *aim* (to say nothing of his intention) had been good, the superintendent might have inflicted a dangerous, perhaps fatal, wound, with this spittoon.

There is another view of it; the State makes appropriation for furniture for this institution, but so far as we know, there is no provision for the purchase of spittoons for belligerant or *throwing purposes.*

We are not informed of the particulars of Dr. Johnson's "charges," but the *Statesman* says they allege "general mismanagement." It is, perhaps, unfortunate that Dr. Johnson is an unsuccessful place-seeker at the Asylum,—a fact which will doubtless be used for all it is worth. But there is perpetual complaint of something wrong at the Asylum, and the subject has gained an unpleasant newspaper notoriety. Dr. Dorsett owes it to himself, and to the State, and to the people, to ask a thorough investigation. If the charges are true, the duty of the Executive is clear, in the premises; if false, Dr. D. should be able to prove their falsity, and by an emphatic disproval forever put the matter at rest; the people are tired of it; and the absence of complaint in the future will be the best evidence that can be adduced in his support. Shall we have it? If trivial, or brought through any unworthy motive, the Board should not entertain the charges. But, in any event, whether Dr. D. asks or desires it, the public demand an investigation, and an impartial and thorough one, and are in no temper to be put off with anything else. The honor and dignity of the State, and the welfare of these unfortunate people, are considerations paramount to any personal rights, privileges or benefits. Where there is so much smoke there must be some fire. The rumors have scarcely died out, of this Superintendent's having dismissed as cured, within a few weeks after his taking charge, one of the most violent and dangerous maniacs in the State—one whose re-arrest and re-committal is said to have cost the county some fifty dollars. Charity seems to have thrown the mantle over that matter, and the people hoped for better things; hence, they were not quite prepared for the spittoon episode, however "justifiable" the honorable Board may be pleased to pronounce it: another "mantle of charity" quite misplaced.

It may be asked—why has not some person moved in this matter before, and brought about an investigation? The reluctance that most men have to becoming informers, as well as the indisposition to antagonise the administration has no doubt, been the restraining influence. Right here, we will do Gov. Ross the justice to say, that Dr. Dorsett's appointment was made, *on as strong endorsements as any man could furnish*, and that personally, the Superintendent was unknown to the Governor.

The following editorial from the *Statesman* of August 7th, is to point, and expresses ours, and the public's sentiments on this subject:

THE LUNATIC ASYLUM.

Now that Dr. Johnson has publicly preferred charges against Dr. Dorsett, superintendent of the insane aylum, it is a fitting opportunity to set at rest the truth or falsity of the various and disparaging rumors concerning the management of the asylum, that have been bruited about for some time past. It has come to that point, where the superintendent himself ought to demand, as the public now demands, a full, complete and impartial investigation. This the board of managers have no right to deny or consent to postpone, nor can they afford to do so in the present inquiring state of public opinion.

There is no doubt, that since the day Dr. Dorsett took charge of the insane asylum, either some one or some class of people have been ceaselessly hounding at his heels, if all these rumors of mismanagement are false; or, if true, that there is a screw loose somewhere in the management that needs tightening. It matters not now whence the rumors have come ; they have gained publicity, and the public will form its decision either with or without facts, and the board of managers of the asylum owe it to the institution to sift these rumors down to the facts and either dispel the present cloud of suspicion, or, if found true, at once correct the abuses. For the question of mal-administration of the asylum has been sprung upon the public, and it should be settled for the sake of the many hundreds of helpless unfortunates in the asylum, if on no other grounds. When the investigation of the asylum does occur we want no star chamber business, but the bottom facts laid before the people of Texas, to whom the asylum belongs, and who have the right to know all the details concerning its management. If the present superintendency is a failure, let it be announced by competent authority. If the prosecution by Dr. Dorsett in this and other rumored matters is groundless, or malicious, let it also be known and the persecutor properly dealt with. Let strict justice be done all around, and, until the facts are all known, judgment should be withheld.

